# The Patentability of Biomolecules – Does Online Bioinformatics Compromise Novelty?

**DOI:** 10.1002/cfg.149

**Published:** 2002-04

**Authors:** Anton Hutter

**Affiliations:** Appleyard Lees Chartered and European Patent Attorneys 15 Clare Road Halifax HX1 2HY UK

## Abstract

Researchers are becoming increasingly concerned that the confidentiality of their novel biomolecule sequences is being jeopardised, particularly when these sequences are either submitted to sequence databases or uploaded as query terms onto internet-based bioinformatic software suites. The researcher's fears stem from the fact that the actual uploading of their sequences acts as a novelty destroying prior disclosure or publication, and that this may subsequently preclude valid patent protection for the sequences. This article addresses the key issues involved in the analyses of biomolecules, highlighting potential risks taken by many researchers in regard to patent protection and suggests possible ways in which these risks may be mitigated.


**Comparative and Functional Genomics**
*Comp Funct Genom* 2002; **3**: 119–126.
Published online 1 March 2002 in Wiley InterScience (www.interscience.wiley.com). **DOI:** 10.1002/cfg.149

**Review**

### The patentability of biomolecules – does online bioinformatics compromise novelty?

Anton Hutter*
*Appleyard Lees, Chartered and European Patent Attorneys, 15 Clare Road, Halifax, HX1 2HY, UK*

**Correspondence to:*
*Appleyard Lees, Chartered and European Patent Attorneys, 15 Clare Road, Halifax, HX1 2HY, UK.*
*E-mail:*
*anton.hutter@applees.co.uk*

Received: 23 January 2002
Accepted: 6 February 2002

### Abstract

**Researchers are becoming increasingly concerned that the confidentiality of their novel biomolecule sequences is being jeopardised, particularly when these sequences are either submitted to sequence databases or uploaded as query terms onto internet-based bioinformatic software suites. The researcher's fears stem from the fact that the actual uploading of their sequences acts as a novelty destroying prior disclosure or publication, and that this may subsequently preclude valid patent protection for the sequences. This article addresses the key issues involved in the analyses of biomolecules, highlighting potential risks taken by many researchers in regard to patent protection and suggests possible ways in which these risks may be mitigated. Copyright © 2002 John Wiley & Sons, Ltd.**

**Keywords: Biomolecule; Bioinformatics; gene sequence; patent; confidential; novelty; disclosure; Internet; e-mail**

### The requirements of patentability in Europe

In order to obtain a valid European patent for any invention, the invention must be novel (Art.54 European Patent Convention; EPC), exhibit an inventive step (Art.56 EPC), and be susceptible of industrial application (Art.57 EPC). An invention is regarded as being novel if it does not form part of the state of the art. The state of the art consists of everything made available to the public anywhere in the world prior to the filing date of a European patent application. An invention is taken to involve an inventive step if, when judged by a person skilled in the art at the time of filing the patent application, it is not merely an obvious development of the state of the art. Finally, an invention is considered as being susceptible of industrial application if it can be made or used in any kind of industry, including agriculture.

In order to obtain a patent directed to a newly discovered biomolecule, for example, a sequence or partial sequence of a gene or protein, one facet of novelty is that the biomolecule must be in an isolated form (Art.5(2) European Biotechnology Directive; BD). In addition, in order to meet the industrial applicability requirement, a specific utility for the sequence or partial sequence which is beyond the realm of mere speculation must be disclosed at the filing date of the patent application (Art.5(3) BD). Hence, it is determining which specific parts of a genome constitute functional genes, and what the functions of those genes are, that may lead to patentable inventions.

At a time when the genome projects of numerous organisms are nearing completion and, in many cases, where the genomes have been fully sequenced, a large amount of biomolecular data is being constantly generated at an incredible rate. Consequently, researchers are filing patent applications for all types of biomolecules in an attempt to protect their intellectual property stemming from this mass of genomic data. In order for these inventions to form the subject of valid patents, they must meet all the requirements of patentability mentioned above. Therefore, when assessing whether a biomolecule is patentable, one of the first questions that must be asked is: 'Is it new?' The question of novelty is very strict and, as part of the requirement for novelty, the biomolecule must be confidential when a patent application in respect of that biomolecule is filed. If the sequence of the biomolecule

Copyright © 2002 John Wiley & Sons, Ltd.

has been publicly used or disclosed, or made available to the public in any way whatsoever prior to the filing date of the patent application, then its novelty may be in doubt. Hence, the validity of the prospective patent may be at risk.

As part of the process of genome research, specifically genome sequencing, the nucleic acid sequences of newly discovered genes are normally made publicly available as cDNAs, via online databases such as those of the European Molecular Biology Laboratory (EMBL), and GenBank. In addition, newly discovered proteins are often published via online databases such as Swiss-PROT and the Martinsried Institute for Protein Sequences (MIPS). GenBank, the EMBL Nucleotide Sequence Database and the DNA Databank of Japan (DDBJ) provide those submitting a sequence to their database with the option of specifying that the sequence data is not added to the online database, and is held confidential for a specified period of time (a 'holding period'). For example, the submitter may require that the sequence is added to the online database only after the sequence has been published in a journal, or after a patent application has been filed in respect of that sequence, thereby maintaining its confidentiality. Around 70–80% of new sequences submitted to the EMBL nucleotide database are subjected to such a confidential holding period and neither the EBI nor the EPO regard this as a novelty destroying public disclosure (Peter Stoehr, personal communication).

Prior to filing a patent application directed to a biomolecule such as a nucleic acid or protein, it is often advisable to investigate the novelty of the sequence of that biomolecule to avoid filing an application for a biomolecule that has already been publicly disclosed, possibly via one of these online databases, or otherwise. The European Patent Organisation (EPO) provides various search facilities for biomolecules by which these investigations may be carried out (**http://www.european-patent-office.org/dg1/ssp/html**). The EPO website includes a clear statement that sequence searching carried out by the EPO is strictly confidential and therefore does not risk the novelty of the sequences searched in terms of patentability.

The purpose of this paper is to investigate the novelty of biomolecules forming the subject matter of patents and patent applications. Specifically, it analyses the use of online bioinformatics software by researchers to determine putative structure and function of their newly sequenced biomolecules, and whether such use online amounts to a prior public disclosure of that biomolecule. As noted above, such a public disclosure may well invalidate a later filed patent application directed to that biomolecule.

### Biology using The Internet

Online bioinformatics is carried out via a network of computers as illustrated by Boxes A-D in Figure 1. The sequence of a biomolecule which, for the purposes of this paper, is a newly sequenced nucleic acid denoted by the term SEQ ID, is stored on a researcher's computer, or client (Box A). For example, SEQ ID may be a gene encoding a novel polypeptide and is regarded as being confidential since it is within the confines of the researcher's computer. In addition, it is assumed that the sequence has not been published in an academic paper, publicly used or disclosed to third parties by the researcher in any way. Hence, while the sequence is still on the researcher's computer, it is still novel in terms of the patenting requirements. In an attempt to determine whether SEQ ID is expressed, the researcher's next step might be to upload the sequence as a query term for subsequent bioinformatics analyses using the wide range of structural and functional predictive software which are currently freely available online via a software server (Box C), for example, the National Centre for Biotechnology Information (NCBI), the Human Genome Mapping Project – Resource Centre (HGMP-RC), or the European Bioinformatics Institute (EBI) etc.

In the case of large pharmaceutical companies and universities, the researcher's computer is able to contact the bioinformatics software server directly via an in-house Internet gateway. However, in the case of smaller organisations, or in circumstances where the researcher accesses the software server online from home, the researcher's computer contacts the software server via a commercial Internet Service Provider (ISP) (Box B), for example, Demon or AOL etc. In the latter case, the query term is transferred to the ISP as a single packet of data via a telephone line shown as path (i). The query term is then divided up and sent as a number of packets of data by the commercial ISP along path (ii) to the software server where the packets of data are reassembled to form the complete query term, SEQ ID. The server, which provides

Copyright © 2002 John Wiley & Sons, Ltd. 

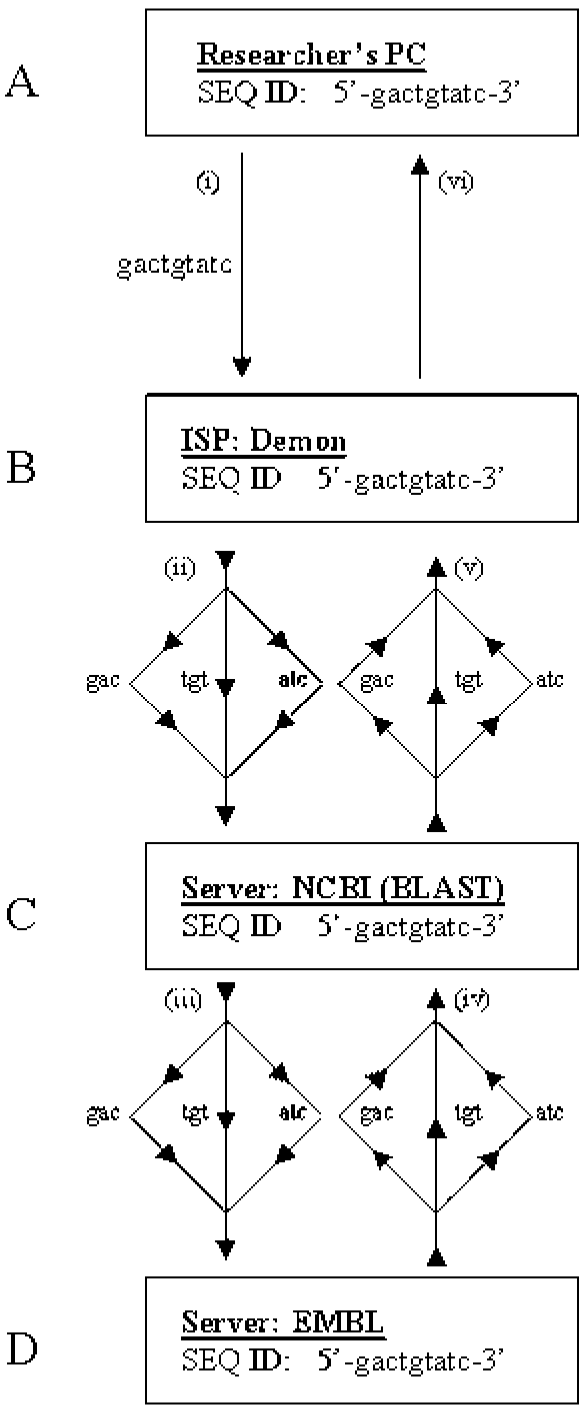

**Figure 1.** A schematic flowchart illustrating a network of computers during the use of bioinformatics software online. A) The researcher's computer on which the novel sequence is saved. B) A local ISP, e.g. demon, AOL etc. C) A server providing bioinformatics software online, e.g. NCBI, Wu-BLAST. D) A server database against which the query term is searched online, e.g. EMBL

bioinformatics software such as the Basic Local Alignment Search Tool (BLAST) program, then divides the query term up into a new set of data packets and sends these along path (iii) to another server (Box D). This second server, for example, EMBL, provides an external database of sequences against which the query term, SEQ ID, may be searched.

Hence, by carrying out bioinformatics analysis of SEQ ID online via the internet, the researcher has voluntarily uploaded the confidential query term from his computer (Box A) onto a bioinformatics software server (Box C), and, therefore, permitted the software server to search the sequence against a sequence database (Box D). The format of results generated by bioinformatics analysis varies widely depending on the predictive software and server used, but in the case of the BLAST program, in most cases the results consist of a detailed list of homology scores and alignments with other sequences in the database, which together may be used to infer putative structure and function of the query sequence, SEQ ID. Such data generated *in silico* is often included in patent applications in an attempt to meet the utility requirement mentioned above. The results are delivered back to the researcher from Box D to Box A along paths (iv), (v) and, in some instances along path (vi), using any of three possible methods available depending on the predictive software and server used. The three methods for receiving the results of bioinformatics analysis carried out online are as follows:-

### Method 1

The search results may be viewed online at a unique temporary Uniform Resource Locator (URL) implemented by the software server. For example, when using the interactive results option with the BLAST program provided by the EBI server, the researcher's BLAST search is assigned a unique 18 digit directory path to where the results of the search are automatically but temporarily posted, and may be viewed online as a hypertext document when the analysis has completed. The search results are maintained online at this unique URL normally for a period of about 24 hours; however, some large files are deleted after about only 15 minutes. Regardless of how long the URL is maintained online, it is possible to view it, and the results, from

Copyright © 2002 John Wiley & Sons, Ltd.

any PC, providing the correct URL is inserted into the web address window of a web browser.

### Method 2

The search results are viewed online at a generic temporary URL generated by the software server. For example, when using the BLAST program provided by the NCBI server, the researcher's sequence is automatically assigned an individual 18 digit request identifier (ID) number shown in an ID window, and then entered into a queue awaiting analysis. Upon completion of the BLAST analysis, the researcher must then press a 'Format' button to view the results. Using the ID number, the server then automatically posts the results of the search to a temporary generic URL, linked to NCBI, which may be viewed online by the researcher. The server also provides the option of viewing the results of different BLAST searches carried out earlier using the NCBI server online by entering a different valid request ID number into the ID window and viewing these results at the same generic URL. A valid ID number is one which has been assigned to a recent BLAST search using NCBI, and consists of data which is still stored in a temporary buffer and which is therefore still available online. As with Method 1, it is possible to view the URL and the results from any PC online, providing a valid ID number is inserted into the ID window.

### Method 3

Some servers provide the researcher with the option of having the results delivered directly back to them via e-mail. For example, when using the BLAST program provided by the IGH Montpellier server, the researcher is given the option of entering his or her private e-mail address into the relevant field prior to carrying out their bioinformatics analysis. Once their e-mail address has been noted, analysis is carried out with the subsequent results being sent to the researcher shortly thereafter.

### Discussion

When assessing whether a biomolecule which has been subjected to online bioinformatics analysis meets the novelty requirement for valid patent protection, one needs to determine whether, in doing so, that biomolecule has been 'made available to the public' in terms of Art.54 EPC. Information is said to be made 'available' to the public if only a single member of the public is in a position to gain access to it and understand it, and if there is no obligation to maintain secrecy. If a person who was able to gain knowledge of an invention was under an obligation to maintain secrecy, the invention cannot be said to have been made available to the public, provided that person does not breach that obligation. In addition, for a disclosure to be novelty destroying, it must be an 'enabling disclosure'. This means it must disclose information in sufficient detail such that the invention may be put into practice by a skilled technician. For example, in the case of a patent for a biomolecule, to be novelty-destroying, the disclosure would have to give details of the biomolecule's sequence and suggest at least one plausible function.

Finally, how an invention is actually made available to the public is in fact immaterial. The EPO Board of Appeal has held that the *theoretical* possibility of having access to an invention renders it in the public domain and, therefore, publicly available, whatever the means by which the invention was made accessible. For example, the EPO Board of Appeal has taken the view that if a document in a library 'would have been available to anyone who requested to see it' on any particular day, then this was sufficient to establish that the document had been made available to the public on that day. It is not necessary, as a matter of law, that any member of the public would actually have been aware that the document was available on that day, or that any member of the public had actually taken note of it. Therefore, it is not that someone *would* view an unreferenced document in a library, the fact that they *could* have viewed it, perhaps even by chance by walking down a random aisle and viewing a random book on a randomly chosen shelf, may be sufficient to result in a novelty destroying prior disclosure. Awareness, or actual inspection, of the document and, hence, invention, does not need to be proven in order for the disclosure to be novelty-destroying. With this, and online bioinformatics analysis, in mind, one needs to ascertain whether any of the three available methods for viewing the results of such analysis (Methods 1, 2 or 3), would result in those results having been 'made available to the public'.

In 2001, the World Intellectual Property Organisation (WIPO) Standing Committee on Patent Law

Copyright © 2002 John Wiley & Sons, Ltd.

conducted a survey on current national and regional practices in Internet-related issues, in particular with regard to publications on the Internet and the security of material contained within e-mails. The relevant document, available at (**http://www.wipo.int/scp/en/documents/session_5**), is SCP/5/4. The general consensus was that any website which may be freely viewed and inspected online by third parties over the internet is regarded as a publication, and that any information contained in a web site prior to the filing date of a patent application acts as fully citable prior art. From the survey results, it appears that the relationship between the general availability/accessibility of a website and it's effect as prior art highlighted a number of conflicting views between the countries who took part. Whilst most countries thought that the possibility of finding a website disclosing an invention using a search engine should be taken into account, some countries believed the degree of difficulty to access the content of any disclosure, be it online or otherwise, should not be relevant. Concerning the duration of the website online, the consensus was that the information should appear on the Internet long enough such that it could be deemed to have been made 'available to the public', this being judged on a case by case basis. On this issue, more than one country stated that, once the information was posted on a website, it was a novelty-destroying disclosure irrespective of the length of its appearance on the Internet.

Hence, it appears that a publicly accessible website is analogous to a book or journal which may be found and read in a library, perhaps only for a limited period of time, but long enough for someone to physically find it in the first place. Therefore, if an invention is described on a live and freely available URL prior to the filing date of a patent application directed to that invention, and with an enabling disclosure, then that website may well be regarded as being a novelty-destroying disclosure thereby invalidating that patent application. To continue the analogy, posting the results of a BLAST search to a URL as in either Method 1 or 2 may be described as being the equivalent of placing a book disclosing the BLAST results in a library without referencing it first, which would still render it publicly available in terms Art.54 EPC. Theoretically, anybody could find the URL and gain access to the search results and the invention.

Using Method 1, anyone could compose a web address for a server hosting bioinformatics software (e.g. **http://www.ebi.ac.uk/**), and a directory path (e.g. servicestmp), which may link to a page of bioinformatics results. Each of these parts of the web address are easily obtainable by simply exploring the software server's website and determining approximately where results are posted. One could then randomly enter digits constituting the file name (e.g. 1234.html) until a valid URL showing a recent set of search results was found. It is appreciated that this may be a very time-consuming process since the odds of finding a valid URL could be as low as 1 in $10^{17}$. Moreover, because the URL showing the results is only available online for a limited period of time, perhaps only a few hours or so, then the time during which a URL address is actually valid is relatively short. In view of the survey results mentioned above, the accessibility of the website, or the virtually 'inaccessibility' thereof, may be a major factor in deciding whether it can be said to have been made available to the public or not. Unfortunately, the absence of legal precedents in this area makes it impossible to conclude whether a website, which is only online for a few hours and incredibly difficult to find, would amount to a prior disclosure. Nevertheless, theoretically, a valid URL could be found, perhaps by using a powerful number-generating algorithm, and it is this theoretical possibility which may well amount to publication of the URL in the eyes of the Courts. This would be analogous to findings the Courts have made in paper-based situations. Therefore, a patent application directed to data disclosed on an online website as in Method 1 could be found to be invalid for lack of novelty.

Using Method 2, anyone is able to freely visit the generic website hosted by the web server which has the ID window and in which a search ID number may be inserted. One could then insert ID numbers until a valid one was found and thereby gain access to the URL showing a recent set of search results. As with Method 1, the likelihood of finding a valid ID number linked to a URL is incredibly slim. However, theoretically, it is possible and, similarly, such a method of viewing search results could be seen to be a publication and therefore novelty destroying. However, in contrast to using Method 1, when using Method 2, an additional step has to be taken in order to view a URL showing bioinformatics results. Whereas in Method 1, one only has to insert the digits directly into the web browser URL window in the hope that a valid website is found immediately, in Method 2, one must insert

Copyright © 2002 John Wiley & Sons, Ltd.

digits into the ID window and then press the 'Format' button before being linked to a URL. Therefore, using Method 2, it is unlikely that any third party attempting to access the results website will have acted in good faith, since they could not accidentally stumble upon it by a 'simple' typographical error.

Finally, using Method 3, the researcher's results are sent from Box C to Box A in Figure 1 via e-mail. The survey conducted by the WIPO Standing Committee on Patent Law also questioned the security of material contained within e-mails and, of the 27 countries that responded on this issue, none stated that they regarded a private e-mail as constituting a public disclosure. This is irrespective of whether encryption is used, and regardless of any warning of confidentiality which may be attached, although it does assume that the recipient is bound by a duty of confidence. The general view is that an e-mail is analogous to a letter or post-card which is consigned to a postal delivery service. A postal employee who sees the letter is not considered to be a member of the public, and should not use the information contained within the letter. Similarly, an employee of an ISP is not considered to be a member of the public and is therefore not free to disclose the information they see in an e-mail, even if there is no bar on employees of ISPs looking at the content of an e-mail as it passes through their servers.

The EBI do not regard the submission of a biomolecule sequence as a query term to their bioinformatics software as having been made publicly available (Peter Stoehr, personal communication). The query sequence and the results are temporarily stored by EBI, but are both deleted shortly after the search has been completed. The sequence and search results are not inspected at all by the database and software support staff except for the purpose of executing the user search and providing user support, if necessary. Every effort is made to keep such temporary data storage confidential and as secure as possible so that neither the query sequence nor the results of the search are made available to the public in any way. Therefore, it would appear that neither the uploading of a query term onto the EBI software server nor the relaying of the results back to the researcher by e-mail are novelty destroying acts *per se*. For a third party to gain access to a temporarily stored query term, or the results of a search, it would have to be by some form of deliberate subterfuge. Intercepting confidential information along any of the paths (i) to (vi) in Figure 1 may be possible using today's hacking methodologies. However, it is more likely to occur when the information is temporarily stored at either of boxes B, C or D where the sequence of the biomolecule consists of a single packet of data instead of a number of separate packets of data as in paths (ii) to (v) which may be difficult to correctly reassemble in to SEQ ID.

### Non-prejudicial disclosures

Unfortunately, things can and do go wrong. A postal worker, sequence database employee or ISP employee may read a letter or e-mail describing an invention and publicly disclose what they have seen. Alternatively, a computer hacker may intercept confidential information describing an invention along paths (i) to (vi) shown in Figure 1 using a 'packet sniffer', and disclose that information to third parties. It is evident that, in all cases, the novelty of the invention and patent application will be put at risk through no direct fault of the patentee. Fortunately, if any of these circumstances do arise, it is possible that such actions would be viewed by the Courts as a breach of confidence by the employees or being unlawfully obtained by a hacker, and therefore be regarded as non-prejudicial disclosures. European patent law accounts for two scenarios in which a prior disclosure of an invention does not prejudice the novelty of a subsequent patent application for that invention, providing the prior disclosure occurred no earlier than six months preceding the filing date of a European patent application (Art.55 EPC). The first scenario is if the patentee had displayed the invention at an official international exhibition or conference. The second scenario is if the disclosure was due to an evident abuse in relation to the patentee, which would include the breach of confidence or unlawful obtaining of information described above. Providing either of these two scenarios occur, there is a six month period of grace in which a valid patent application may still be filed.

In order to take advantage of Art.55 EPC, the patentee needs to show that an evident 'abuse' had occurred to their detriment. It has been suggested that deliberate intention to harm another party may constitute evident abuse, as would knowledge of the possibility of the harm resulting from a planned

Copyright © 2002 John Wiley & Sons, Ltd. 

breach of confidentiality. Therefore, one must determine whether the hacker or database/ISP employee intends to harm the patentee by carrying out an act of disclosure. It is possible that the same consideration may be applied to the case where an individual intentionally attempts to access a unique URL showing results from Method 1 or Method 2. In this case, someone could locate a results page by the random insertion of digits into the URL window (Method 1) or ID number (Method 2). Such an act could be seen as on a par with attempting to view a website which is only accessible by a secret password, and therefore as an 'abuse' in accordance with Art.55 EPC. Unfortunately, without the benefit of case law to help guide practitioners, it is difficult to say whether abuse has been committed, and each of these scenarios would have to be judged on a case-by-case basis.

Therefore, if Art.55 EPC is to come into play, it is important to ensure that the employees or hacker, who may see the query sequence, or read the e-mail/letter by accident or intentionally, and who may subsequently disclose the material contained in it, clearly understands that he is acting unlawfully and/or is bound by a duty of confidence. Unfortunately, the websites of EBI (and other database and software providers) do not include a clear statement indicating that they regard all information as being confidential. Therefore, a clear warning alerting the reader to the confidentiality of any material, be it either a sequence uploaded onto a server, or bioinformatics results being posted on to a webpage, or being sent to the researcher via e-mail may increase the chances of Art.55 EPC being applied.

### Conclusion

Having examined the various methods by which the results of online bioinformatics analysis may be viewed or returned to the researcher, it is now possible to categorise these alternatives according to their risk of constituting a novelty destroying publication under Art.54 EPC. Of the three methods discussed, it is the author's view that having the results returned via e-mail is likely to be the option involving the least risk (Method 3). The general consensus is that e-mails are confidential communications between a sender and a recipient and are not viewed as public disclosures. However, not all servers provide the option of returning results by e-mail. The method posing the next least risk is when the software server assigns a unique user ID search number which is used to view a URL at which the results are temporarily posted (Method 2). It is the author's view that this method is less likely to be seen as a publication because of the extra step which must be taken by the researcher (or third party) before activation of the results website online. Using Method 1, where the results are automatically posted onto a website, and where, in theory, anyone could potentially see the website by accident, probably poses the highest risk in respect of acting as a publication, and it is suggested that this method should be avoided if at all possible.

With all three methods, if a prior disclosure occurs as a result of abuse by third parties who have intercepted the online or e-mailed results, then it may still be possible to file a patent application with a valid claim to novelty, providing the application is filed within six months of that prior disclosure, by taking advantage of Art.55 EPC. To facilitate this, it is suggested that online software servers and online databases include a clear secrecy declaration stating that users' query terms and results are regarded as strictly confidential, so that any breach of this confidence could be asserted to be evident abuse.

It is interesting to speculate that if the Courts adopt the view that results of bioinformatics analyses carried out over the web which are posted at an online website, albeit a temporary one, do act as public disclosures (Methods 1 or 2), then we could have a rather awkward situation in which a patent directed to an online-researched biomolecule may actually lack novelty and, therefore, be invalid. If this were to be the case, one wonders how many patents may be invalid because they cover biomolecules which used online bioinformatics prior to filing. Many large bioscience research organisations adopt the policy of maintaining 'local' or 'in-house' sequence databases, and carry out all their bioinformatics behind a secure firewall. The advantage of this system is that there is no need to conduct research online thereby avoiding any of the aforementioned risks associated with working over the Internet. Hence, it is the author's view, that this method is the most risk-free manner in which bioinformatics research could be conducted, and therefore cannot be recommended strongly enough where cost is not a major issue. Disadvantages of this system include the necessity for incredibly large hard-drives on which the sequence databases and bioinformatics software must be downloaded and

Copyright © 2002 John Wiley & Sons, Ltd.

fully supported. Furthermore, it is necessary to continually update the sequence databases, normally on a daily basis, in order to avoid them becoming out of date.

Most smaller organisations, such as biotech start-up companies and many universities, do not have the considerable financial reserves required to set up an in-house computing facility and manage query tool support locally. As a result, it is entirely possible that these organisations have conducted, and are currently conducting, research over the web, thereby disclosing their biomolecules before the filing date of a patent application.

Finally, if online bioinformatics is viewed as acting as a novelty destroying prior disclosure, it may be possible to take advantage of a twelve month period of grace which is available for filing patent applications in the United States. If an invention has been disclosed anywhere in the world, for example on a website, then a patent application may still be filed with a valid claim to novelty in the US providing it is filed no later than twelve months after that disclosure. It should be noted that this grace period may only be used when filing for protection in the US. When valid patent protection is sought in countries other than the US, it is almost always necessary to ensure that an invention has not been disclosed prior to the filing date.

The above relates to the author's views on the potential risks inherent with using online bioinformatics, and should not be taken as legal advice for any particular circumstances. If specific advice is required, it is recommended that a Chartered or European patent attorney be contacted.

### Acknowledgement

Thanks go to the staff of Appleyard Lees, Peter Stoehr of EMBL-EBI, Mike Barnes of GlaxoSmithKline, Chris Southan of Gemini Genomics, and Jo Wixon, Managing Editor of CFG for stimulating discussions and support during the writing of this paper.

Copyright © 2002 John Wiley & Sons, Ltd.

